# Detection and Modulation of Olfactory Sensing Receptors in Carnivorous Rainbow Trout (*Oncorhynchus mykiss*) Fed from First Feeding with Plant-Based Diet

**DOI:** 10.3390/ijms23042123

**Published:** 2022-02-14

**Authors:** Cécile Heraud, Théo Hirschinger, Elodie Baranek, Laurence Larroquet, Anne Surget, Franck Sandres, Anthony Lanuque, Frederic Terrier, Jérôme Roy

**Affiliations:** INRAE, E2S UPPA, UMR1419 Nutrition Metabolism and Aquaculture, Aquapôle, Université de Pau et des Pays de l’Adour, F-64310 Saint-Pée-sur-Nivelle, France; cecile.heraud@inrae.fr (C.H.); theo.hirschinger@inrae.fr (T.H.); elodie.baranek@inrae.fr (E.B.); laurence.larroquet@inrae.fr (L.L.); anne.surget@inrae.fr (A.S.); franck.sandres@inrae.fr (F.S.); anthony.lanuque@inrae.fr (A.L.); frederic.terrier@inrae.fr (F.T.)

**Keywords:** alternative protein source, chemosensory receptor, odorant sensing receptor, taste receptor, farmed fish

## Abstract

Sense of smell is mediated by diverse families of olfactory sensing receptors, conveying important dietary information, fundamental for growth and survival. The aim of this study was to elucidate the role of the sensory olfactory pathways in the regulation of feeding behavior of carnivorous rainbow trout (RT, *Oncorhynchus mykiss*), from first feeding until 8 months. Compared to a commercial diet, RT fed with a total plant-based diet showed drastically altered growth performance associated with feed intake from an early stage. Exhaustive examination of an RT genome database identified three vomeronasal type 1 receptor-like (ORA), 10 vomeronasal type 2 receptor-like (OLFC) and 14 main olfactory receptor (MOR) genes, all highly expressed in sensory organs, indicating their potential functionality. Gene expression after feeding demonstrated the importance in olfactory sensing perception of some OLFC (*olfcg6*) and MOR (*mor103*, -*107*, -*112*, -*113*, -*133*) receptor family genes in RT. The gene *ora1a* showed evidence of involvement in olfactory sensing perception for fish fed with a commercial-like diet, while *ora5b*, *mor118*, *mor124* and *olfch1* showed evidence of involvement in fish fed with a plant-based diet. Results indicated an impact of a plant-based diet on the regulation of olfactory sensing pathways as well as influence on monoaminergic neurotransmission in brain areas related to olfactory-driven behaviors. The overall findings suggest that feeding behavior is mediated through olfactory sensing detection and olfactory-driven behavior pathways in RT.

## 1. Introduction

Most fish species are endowed with an exquisite olfactory sense that enables them to gain important environmental information to detect the proximity of food, conspecifics, mates or predators and to avoid contaminants [1]. Odorant perception is a multistep process that results from the activation of specific odorant receptors expressed by olfactory neurons interacting with neural circuits in the olfactory rosette of the nasal cavity [2]. The olfactory signal is ultimately processed and translated into specific behavioral outputs, such as regulation of feed intake [3].

Since the first odorant receptor was identified [4], the same classes of olfactory receptor have been found in fish [5]. Olfactory receptors exhibit a predicted seven transmembrane topology and sequence motifs characteristic of G-protein-coupled receptors (GPCR). Subsequent to the discovery of the main olfactory receptor (MOR), two other types of GPCR, ORA (olfactory receptors related to class A) and OLFC (olfactory C) families were described in fish [5]. These olfactory receptors are found on different types of sensory neurons, MOR in the ciliated sensory neurons, OLFC in the microvillus sensory neurons, and nearly all crypt neurons express a single member of the ORA gene family. Regarding rainbow trout (RT, *Oncorhynchus mykiss*), which has major value in the world aquatic species trade, only a few studies have described the anatomy of the olfactory system [6]. 

Information on olfactory sensing receptor regulation associated with diet change is crucial to elucidate the role of the sensory olfactory pathway in the regulation of feeding behavior of RT. This knowledge is also important in formulating feed ingredients for aquaculture, to sustain and promote growth, development, and the health performance of farmed fish. Aquaculture production is expanding by 3–4% annually [7]. The increase in demand for aquafeeds cannot be satisfied by reliance on wild caught fish, which have already reached their limit in capture fisheries. The availability of traditional aquafeed ingredients, including fish meal (FM) and fish oil (FO), has not increased with demand, and today the traditional ingredients of aquafeeds must be replaced by renewable, eco-friendly and less costly alternative raw materials [8]. However, the total replacement of marine products by plant ingredients has not yet been achieved and several bottlenecks remain. A previous study from our laboratory revealed that the total replacement of FM and FO with plant ingredients from first feeding of RT (carnivorous species) leads to reduction in the growth and survival rate of this fish species [9] mainly related to an alteration of feed intake [10]. Therefore, it is essential to improve fish feeding strategies—feeding represents up to 50% of total production costs—both by increasing feed intake and feed efficiency. For twenty years, it has been known that the olfactory perception of food odors by fish, in most cases, precedes the discovery and grasping of the food item and increases their feeding activity [11]. It is still unknown whether olfactory sensing receptor stimulation caused by food odors affects gustatory food properties and, consequently, the recognition and consumption of food by fish. The present study aims to elucidate the role of sensory olfactory pathways in the regulation of feeding in RT fed with a plant-based diet devoid of the high value traditional aquafeed ingredients of FM and FO found in commercial diets. 

We hypothesized that the absence of FM/FO in the new alternative plant-based diet would disregulate feeding behavior in RT through olfactory sensing mechanisms due to variations of some nutrients between the two diets. To achieve these objectives, we compared olfactory sensing pathways in RT fed with two diets formulated and manufactured in our experimental facility, a commercial-like diet (C) or an isocaloric plant-based diet (V), from first feeding until eight months. After in silico identification of olfactory sensing receptors, the study focused firstly on the regulation of olfactory receptors in fish fed for eight months with C or V diets, and secondly on the effect of meals of these two diets on the regulation of these olfactory sensing receptors (with a control condition involving eight months diet followed by fasting for five days). This study, for the first time, assessed the detection of diets after long-term exposure as a result of regulation of olfactory sensing detection and olfactory-driven behavior pathways after a post-prandial period in farmed fish.

## 2. Results

### 2.1. Growth Performance and Feed Parameters from First Feeding to 8 Months

**Figure 1:**
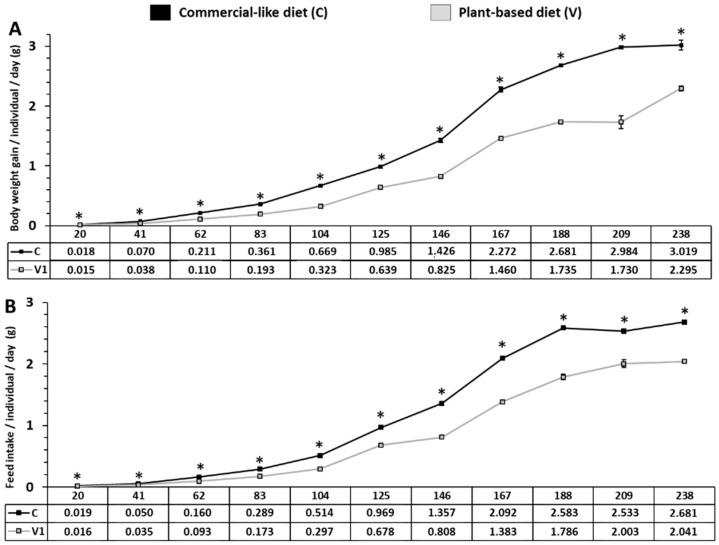
Body weight gain per day and feed intake of RT fed with C vs. V diet from first feeding to eight months. (**A**) Comparison of body weight gain per individual per day by period of 3 weeks for trout fed with C or V diet for eight months. (**B**) Representative feed intake (individual/day) for RT fed with C or V diet for eight months. An asterisk indicates a significant difference between the two dietary treatments as determined by one-way ANOVA (*p* < 0.05). Results are expressed in grams as mean ± SEM (*n* = 5 tanks).

From day 20 to day 238, RT fed with the C diet displayed higher body weight gain (BWG) compared to the V diet (*p* < 0.05; Figure 1A). At eight months, final body weight was 344 ± 3.48 g for fish fed with the C diet vs. 228.9 ± 1.07 g for fish fed with the V diet (*p* < 0.05; C vs. V, data not shown). Daily feed intake was significantly higher for all feeding periods for fish fed the C diet vs. the V diet (*p* < 0.05, Figure 1B). From the third period (day 62) to the eighth period (day 167), daily feed intake was over 150% higher for fish fed with the C diet vs. the V diet. 

### 2.2. Presence of Several Sequences of Olfactory Sensing Receptors in RT

In order to identify putative sequences corresponding to olfactory receptors in the RT, we performed an in silico analysis using a newly available, annotated RT genome assembly, Omyk_1.0 and compared results with data available in other selected species, e.g., human, mouse, salmon and zebrafish (Table 1). For the V1R-like receptor family, three ORA receptors, *ora1*, *ora3* and *ora5* were found in the RT genome. The number of ORA receptors in RT is close to that of salmon (seven) and zebrafish but also to human (four), albeit very low if compared to the mouse (164). Each ORA has two paralogs in the RT genome. For the V2R-like receptor family, 10 OlfC receptors were identified in the RT genome compared to 29 for salmon and 50 for zebrafish, whereas no V2R-like receptors are reported in humans and 121 are found in the mouse. *Olfcr1* and *olfcv* had two paralogs and their sequence homologies were above 95%. For the MOR receptor family, 14 receptors were identified in the annotated RT genome assembly, Omyk_1.0 compared to 388 for humans, 1037 for the mouse, 11 for salmon and 37 for zebrafish. *mor103* had three paralogs (103.2, 103.3 103.4) and *mor129* two paralogs (1a and 1a); their sequence homologies were above 95%.

### 2.3. Expression of mRNAs Encoding V1R-Like Olfactory Sensing Receptors in RT

**Figure 2:**
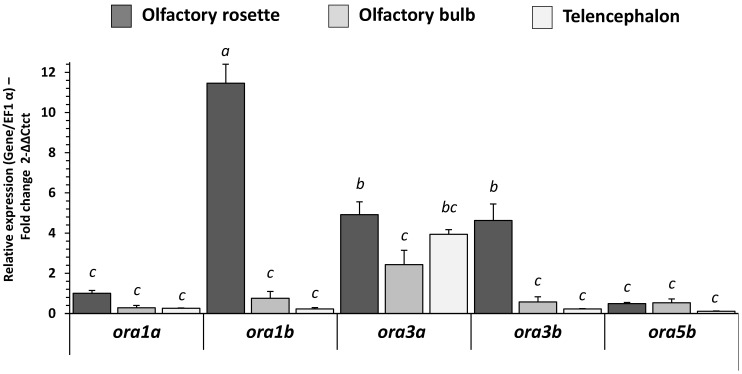
Level of mRNA transcripts of the ORA gene receptor family in olfactory rosette, olfactory bulb and telencephalon of RT fed with C diet for eight months (5 days fasted afterward). Relative gene expression measured by real-time [quantitative] PCR (RT-PCR) of ORA gene family. Values are expressed as group mean ± SEM; fold change 2^−∆∆Ct^ vs. *ora1a* gene C diet (olfactory rosette) for all genes. One-way ANOVA, Tukey post hoc; letters indicate a significant difference (*p* < 0.05) between organs (*n* = 8) or receptors (*n* = 8).

Expression of all mRNAs encoding V1R-like olfactory sensing receptors was detected in all the studied regions of the RT (i.e., olfactory rosette, olfactory bulb and telencephalon; Figure 2), except *ora5a* (not shown). All ORA genes were predominantly expressed in the olfactory rosette, compared to the bulb and the telencephalon, except *ora5b* which was expressed equally in the rosette and the olfactory bulb. The expression level of V1R-like receptor transcripts in the olfactory rosette of fish fed with the C diet showed that *ora1b* was the most expressed, followed by *ora3a* and *ora3b*, then *ora1a* and *ora5b*. *ora1b* was expressed 12-fold more frequently than the paralog *ora1a or ora5b*, and 2.4-fold more than *ora3a* and *ora3b* (with the same mRNA abundance as *ora3*).

### 2.4. Influence of V Diet and Fasting on mRNA Expression Levels of V1R-Like Receptor in RT

**Figure 3:**
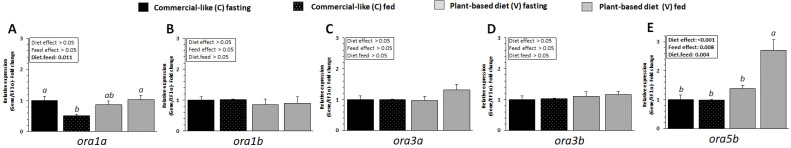
ORA-related mRNA level of transcripts in olfactory rosette of RT fed for eight months with C vs. V diet. Relative gene expression measured by RT-PCR of (**A**) *ora1a*, (**B**) *ora1b*, (**C**) *ora3a*, (**D**) *ora3b* and (**E**) *ora5b* genes after 5 days of fasting and 30 min after feeding. For expression of values and statistics, see legend for Figure 2.

There was no effect of diet, feeding status or their interaction for the expression of *ora1b*, *ora3a* and *ora3b*. However, fish fed with the C diet displayed a 2-fold downregulation for *ora1a* after feeding (Figure 3A; *p* < 0.05), which was not observed in the V dietary group. The expression of *ora5b* was also affected with an upregulation after feeding in the fish fed in the V diet group, but not in the C dietary group (diet × feed interaction, Figure 3E; *p* < 0.05).

### 2.5. Expression of mRNAs Encoding V2R-Like Olfactory Sensing Receptors in RT

**Figure 4:**
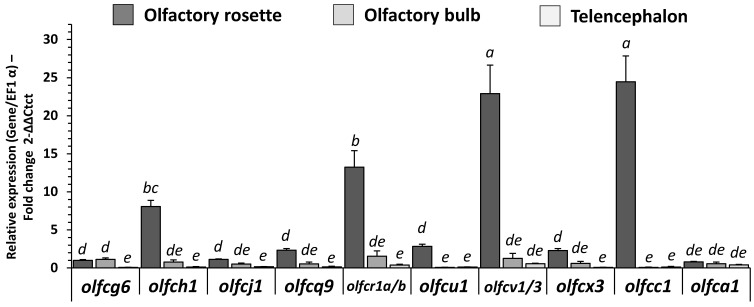
OLFC gene receptor family related mRNA level of transcripts in olfactory rosette, olfactory bulb and telencephalon of RT fed with C diet for eight months (5 days fasted afterward). Relative gene expression measured by RT-PCR of OLFC genes family. For expression of values and statistics, see legend for Figure 2.

The expression of mRNAs encoding V2R-like olfactory sensing receptors was detected in the three studied regions of the RT (i.e., olfactory rosette, olfactory bulb, telencephalon; Figure 4). All OLFC genes were predominantly expressed in the olfactory rosette but also in the olfactory bulb for *olfcg6* and *olfcj1.* Moreover, *olfca1* was expressed in the three studied tissues. Regarding their relative expression in olfactory rosettes, *olfcv1/3* and *olfcc1* were predominantly expressed (more than 20-fold change expression compared to *olfcg6*) compared to *olfcr1a-b*, *olfch1*, with a faint relative expression for other V2R-like receptors.

### 2.6. Influence of V Diet and Fasting on mRNA Expression Levels of V2R-Like Receptor in RT

**Figure 5:**
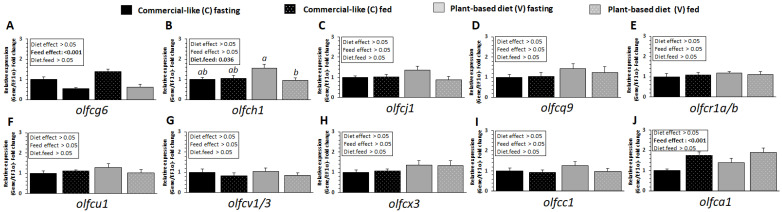
OLFC related mRNA level of transcripts in olfactory rosette of RT fed for eight months with C diet vs. V diet. Relative gene expression measured by RT-PCR of (**A**) *olfcg6*, (**B**) *olfch1*, (**C**) *olfcj1*, (**D**) *olfcq9*, (**E**) *olfcr1a-b*, (**F**) *olfcCu1*, (**G**) *olfcu1-3*, (**H**) *olfcx3*, (**I**) *olfcc1* and (**J**) *olfca1* gene after 5 days of fasting and 30 min after feeding. For expression of values and statistics, see legend for Figure 2.

The expression of *olfcg6* and *olfca1* receptor genes was significantly affected by feeding (Figure 5A,J, feed effect, *p* < 0.05). The mRNA level of *olfcg6* was downregulated after feeding, and upregulated for *oflca1* (Figure 5A,J) for the two diets. In fish fed with the V diet, the mRNA abundance of *olfch1* was significantly increased by fasting compared to fish fed with the C diet (diet × feed interaction *p* < 0.05, Figure 5B).

### 2.7. Expression of mRNAs Encoding MOR Olfactory Sensing Receptors in RT

**Figure 6:**
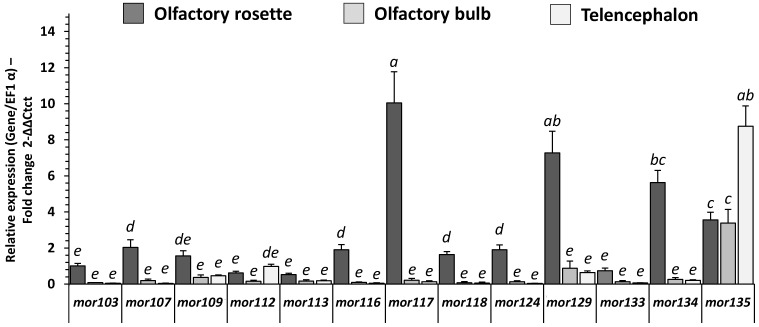
MOR gene receptor family related mRNA level of transcripts in olfactory rosette in ol-factory rosette, olfactory bulb and telencephalon of RT fed with C diet for eight months (5 days fasted afterward). Relative gene expression measured by RT-PCR of MOR gene family.

The expression of mRNAs encoding MOR olfactory sensing receptors was detected in the three studied regions of the RT (i.e., olfactory rosette, olfactory bulb, telencephalon; Figure 6), except for *mor115* (not shown). All MOR genes were predominantly expressed in the olfactory rosette, except for *mor135*, which was predominantly expressed in the telencephalon, and *mor112*, which was equally expressed in the rosette and the telencephalon. The level of MOR receptor mRNA in the olfactory rosette of fish fed with the C diet showed that *mor117* was the most expressed MOR receptor. The transcripts of *mor116*, *mor129*, *mor134*, and *mor135* were also highly detected compared to other MOR receptors.

### 2.8. Influence of V Diet and Fasting on mRNA Expression Levels of MOR Receptor in RT

**Figure 7:**
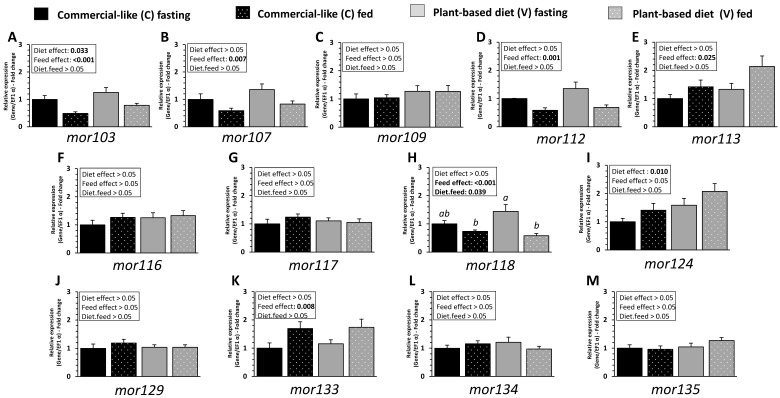
MOR related mRNA level of transcripts in olfactory rosette of RT fed for eight months with C diet vs. V diet. Relative gene expression measured by RT-PCR of (**A**) *mor103*, (**B**) *mor107*, (**C**) *mor109*, (**D**) *mor112*, (**E**) *mor113*, (**F**) *mor116*, (**G**) *mor117*, (**H**) *mor118*, (**I**) *mor124*, (**J**) *mor129*, (**K**) *mor133*, (**L**) *mor134* and (**M**) *mor135* after 5 days of fasting and 30 min after feeding. For expression of values and statistics, see legend for Figure 2.

Expression of *mor103*, *mor107*, *mor112*, *mor113* and *mor133* receptor genes were significantly affected by feeding (Figure 7A,B,D,E,K, respectively, diet effect, *p* ˂ 0.05); being decreased (*mor103*, *mor107)* or increased (*mor113*, and *mor133)* upon feeding. There was no effect of diet, feeding status or interaction for *mor109*, *mor116*, *mor117*, *mor129*, *mor134* and *mor135* (Figure 7F,G,J,L,M, respectively; *p* ˂ 0.05). No difference in mRNA abundance and direction of modulation for each receptor was observed between the two diets, except for *mor118* which was downregulated after feeding only for fish fed with the V diet (diet × feed interaction *p* < 0.05, Figure 7H), and *mor103* and *mor124* whose level of expression was higher for fish fed with the V diet compared to fish fed with the C diet (diet effect *p* < 0.05 Figure 7A,I, respectively).

### 2.9. Influence of V Diet in mRNA Expression Levels of Olfactory and Brain Markers

**Figure 8:**
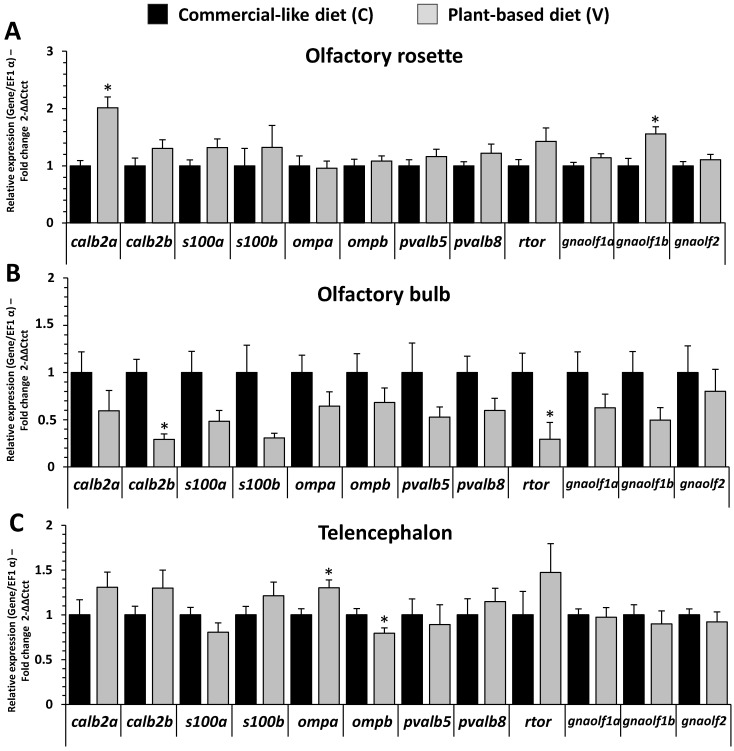
Olfactory and brain marker related mRNA level of transcripts in olfactory rosette, olfactory bulb and telencephalon of RT fed for eight months with C vs. V diet (5 days fasted afterward). (**A**) Relative gene expression measured by RT-PCR of olfactory and brain markers in olfactory rosette. (**B**) Relative gene expression measured by RT-PCR of olfactory and brain markers in olfactory bulb. (**C**) Relative gene expression measured by RT-PCR of olfactory and brain markers in telencephalon. Values are expressed as group mean ± SEM (*n* = 8); fold change 2^−∆∆Ct^ vs. C diet for all genes; asterisk indicates a significant difference between the two dietary treatment as determined by a *t*-test (*p* < 0.05).

In the olfactory rosette, *calb2a*, a marker of olfactory epithelium and ciliated cells, and of *gnaolf1b*, another marker of olfactory cells, were upregulated for fish fed with the V diet (Figure 8A). In the olfactory bulb, *calb2b*, a marker of olfactory epithelium and ciliated cells, and of *rtor*, a marker of odorant receptors, were downregulated in fish fed with the V diet (Figure 8B). In the telencephalon, *omp* (olfactory marker protein) paralog markers of mature olfactory sensory receptor neurons were upregulated for *ompa* and downregulated for *ompb* in fish fed with the V diet (Figure 8C). Expression of all other markers related to olfactory microviliate cell markers (*s100a*, *s100b*) and olfactory tissue markers (*pvalb5*, *pbalb8*) were unaffected by the nature of the diet.

### 2.10. Influence of V Diet on Brain Concentration and mRNA Level of Metabolism Pathways of Catecholamines and Indolamines in Telencephalon of RT

**Figure 9:**
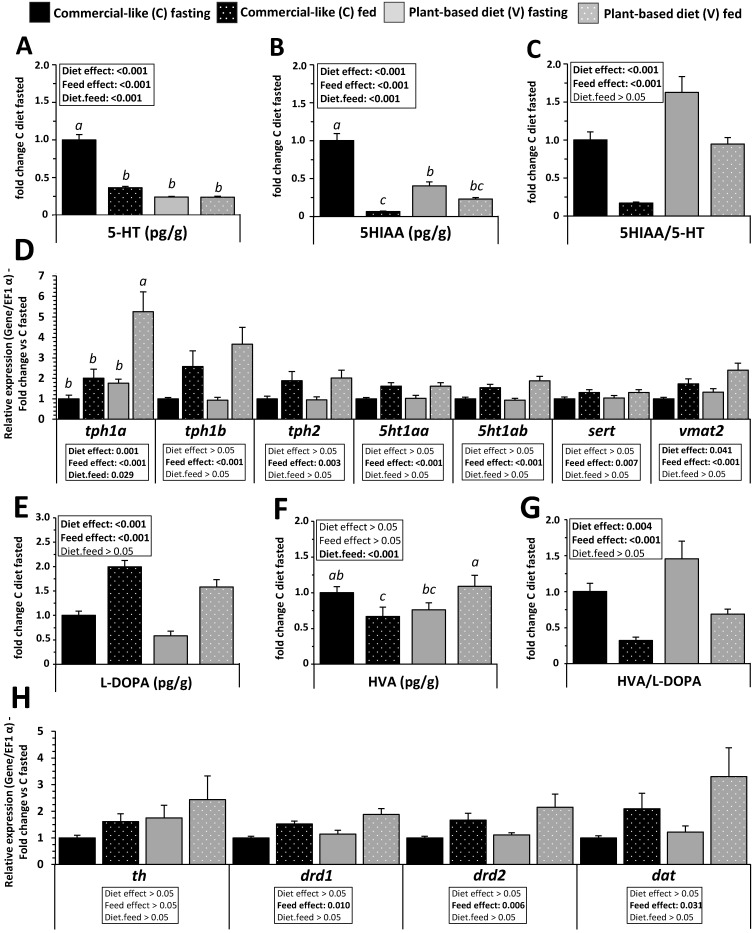
Brain concentrations of indolamines, catecholamines, and corresponding metabolism pathways in telencephalon of RT fed for eight months with C vs. V diet. (**A**–**C**) brain concentration of indolamines (5-HT, 5-HIAA and turnover ratio 5-HT/5HIAA), and (**E**–**G**) catecholamines (L-DOPA, HVA and turnover ratio HVA/L-DOPA). Values are expressed as group mean ± SEM (*n* = 12). Relative gene expression (fold change to C diet for each gene) measured by RT-PCR of metabolic pathways of indolamines (**D**) and catecholamines (**H**). Values are expressed as group mean ± SEM (*n* = 8); two-way ANOVA followed by Tukey post hoc test; if interaction (diet × feed), letters indicate a significant difference between conditions as determined by a one-way ANOVA (*p* < 0.05).

Catecholamine and indolamine brain turnover was mainly affected in the telencephalon of RT fed with the V diet. In the fasting period, indolamine (5-HT, 5-HIAA, Figure 9A,B) concentrations were higher in fish fed with the C diet for eight months than fish fed with the V diet. Indolamines and turnover ratio (Figure 9C) were decreased by feeding of fish fed with the C diet but not for fish fed with the V diet. The 5-HIAA/5-HT ratios in the telencephalon of fish fed the V diet were higher than in fish fed with the C diet after or before the feeding period (diet effect, *p* < 0.05, Figure 9C). The levels of all mRNA trancripts related to the metabolism pathway of 5-HT were upregulated after feeding for both dietary treatments (feed effect, *p* < 0.05, Figure 9D). The abundance of mRNA of *tph1a* (enzyme responsible of 5-HT synthesis from tryptophan) and *vmat* (vesicular transporter of 5-HT) was higher for the V dietary group before or after feeding period or for the diet compared to the C dietary group, respectively (diet effect, *p* < 0.05, Figure 9D). 

Regarding catecholamine concentration, L-DOPA was increased after the feeding period for the two diets (feed effect, *p* < 0.05, Figure 9E), but concentration was higher for fish fed with the C diet for eight months (diet effect, *p* < 0.05, Figure 9E). HVA concentration was lower for fish fed with the C diet, whereas a higher concentration was observed for fish fed with the V diet after the feeding period (diet x feed interaction, *p* < 0.05, Figure 9F). The HVA/L-DOPA ratio in the telencephalon of fish fed with the V diet decreased after feeding for diets (feed effect) being even higher for fish fed with the the V diet (diet effect, *p* < 0.05, Figure 9G). The level of mRNA transcripts related to the dopamine (DA) metabolism pathway was upregulated after feeding with dietary treatments except for *th* the level of which remained unchanged (feed effect, *p* < 0.05, Figure 9H).

## 3. Discussion

As previously observed [9,12,13], a total plant-based diet devoid of FM and FO reduced the growth performance of RT. Interestingly, in our feeding conditions, RT fed with V diet for eight months showed an early alteration of their growth from the first feeding periods, mainly related to alteration of feed intake. To our knowledge, this is the first report which demonstrates such an early impairment of growth and feed intake in RT fed with alternative plant diets. All measures of food intake showed a decrease in the amount of food ingested by RT from the early stage to eight months. Many of the nutritional studies performed on RT to date, which have focused on growth performance, have focused on feed efficiency, while acceptance of feed through molecular integrative pathways, which is the first limiting step determining efficient growth performance, has been neglected. Taste and smell are the first systems involved in sensory detection of nutrients that play a key role in the regulation of feeding behavior and energy balance [14]. They are the main drivers of food approach and choice and are fundamental for diet selection in vertebrates.

For the gustatory sensing system, our previous research assessed the detection and modulation of taste receptors in RT fed with a total plant based-diet, with or without supplementation by ω-3 LC-PUFA [15]. The results of this study showed that RT have the fundamental mechanisms for oro-gustatory perception of nutrients related to different diet composition. In particular, we have provided the first set of evidence suggesting that taste receptors are involved in the oro-gustatory perception of different diets and could play a role in the regulation of feeding behavior.

Feed ingredients (i.e., marine animal versus plant origin) may influence the intestinal epithelial structures and the local immune status in the gut of fish that can affect their metabolism, their ability to digest fatty acids and their food efficiency for different diets [16,17]. Although it is likely that RT have effective LC-PUFA synthesis pathways [18], especially when young, the assimilation and digestibility of PUFA precursors (e.g., ALA, alpha-linolenic acid) in diets containing these (V diet) might not be efficient at the fry stage. For instance, in our study, feeding efficiency was slightly altered (data not shown) at an early stage for fish fed with the V diet. 

Here, we demonstrated that the olfactory sensing system is another major system involved in long-term diet alteration, as shown by modulation in the expression of several families of olfactory receptors in RT and the selection of some of these according to the diet. The olfactory receptors may detect specific food-borne flavor compounds and react to food uptake. Therefore, volatile compounds (i.e., affecting the aroma of food) may contribute significantly to perceived taste affecting the regulation of feeding behavior of RT. Consistent with this view, in silico comparison showed considerable variation in the distribution and number of the olfactory receptors between species. In fish, the distribution of receptors varies between the three main classes with the repertoire being much more important for zebrafish (93) compared to trout (27 in total, see Table 1). 

Our data indicated the detectable expression of almost all the olfactory sensing receptors in the sensory organs of RT, mainly the olfactory rosette, except for a small number, which could suggest they are essential for the proper functioning of odor perception. These differences between species could enable the fine discrimination of closely related odorants present in species-specific nutritional environments. The small number of olfactory receptors in RT (30-fold less than in a mouse) suggests the possibility that, to a greater extent than the phylogeny trait, several extrinsic factors involving olfactory sensing (e.g., finding food, hunger pangs and possibly the need to avoid predators) play an important role in the regulation of feeding behavior in RT [19]. A previous comparative genomic analysis revealed a disparity in the number of receptors between mammals and fish, suggesting an evolutionary shift of olfactory receptor gene repertoires in vertebrates related to the transition from water to land [20]. Although the ligands of most V1Rs and V2Rs are yet to be identified, the available functional data suggest that V1Rs bind to small volatile chemicals, whereas V2Rs bind to water-soluble molecules [21,22]. Thus, aquatic vertebrates encounter and need to detect more water-soluble molecules, whereas land vertebrates need to sense more volatile chemicals. For RT, this interpretation is reasonably valid with 10 V2Rs vs. three V1Rs. For the MOR repertoire, only a few putative MOR genes have been identified in fish and their role remains purely speculative.

In this study, we suggest that the absence or presence of some nutrients, which differ between the V diet and the C diet, modify the regulation of feeding behavior by impacting central activity, including modulation of the sensory olfactory perception and sensing pathways. In particular, lipids, which constitute one of the three main classes of nutrients, along with carbohydrates and proteins, were by far the major source of difference in terms of nutrient replacement in the V diet vs. the C diet. Substitution of FM and FO involves the use of plant ingredients that are free of essential ω-3 LC-PUFAs, mainly EPA and DHA, known to influence the feeding behavior of RT [23]. In our study, the major difference in terms of nutrients was DHA (26.53%) present in FO of the C diet and replaced by alpha-linolenic acid (ALA; 28.24%) in the V diet. As we recently suggested for the gustatory system [15], the difference in the proportion of ω-3 PUFAs between the two dietary treatments could involve the regulation of olfactory sensing receptors influencing the feeding behavior of RT. In this study, some olfactory receptor-encoding genes display modulation of their expression regarding diet and/or feeding status and are found in the three main olfactory receptor families.

For the ORA gene family, the two ORA receptors that were regulated by diets were the ones with the lowest expression in RT after eight months. One major mechanism controlling GPCR responsiveness is the activation-dependent regulation of receptors, also called homologous desensitization [24]. Chronic activation of a receptor leads to a reduced ability to be stimulated in the future, whereas low activation leads to an increased ability to be stimulated. A given dose of agonist or nutrient in our study may give distinctly different responses depending on the prior activation state of the system. This results in over expression of unstimulated receptors and under expression of stimulated receptors. Thus, the lower mRNA level of *ora1a* and *ora5b* olfactory sensing receptors implies their chronic activation by nutrients for eight months suggesting their role in olfactory sensory detection in RT. Moreover, these two genes were differentially regulated by our two diets in the postprandial period. In view of our results, *ora1a* and *ora5b* are two receptors inferred to be involved in the detection of nutrients in RT, especially for water-insoluble lipids. Nevertheless, due to the absence of studies of receptor desensitization in fish, we cannot exclude the opposite hypothesis, that the absence of ligands of these receptors could have led to a decrease in their expression. 

The same hypothesis is applicable to the two other olfactory receptor classes in RT. For the OLFC olfactory receptors family, as compared to ORA, we found significant heterogeneity in their expression in the olfactory rosette. Moreover, some OLFC genes were down and upregulated by feeding with the C and V diets (feeding effect). Some OLFC receptors from the goldfish and zebrafish were activated by amino acids [25], some of which are potent feeding cues in fish. In our study this function could be assumed for *olfcg6* and *olfca1* and *olfch1*. For the MOR sensing receptor, we found significant heterogeneity in their expression in the olfactory rosette. Interestingly, and compared to the V1R and V2R families, our study revealed that many MOR (6/13) genes were regulated by feeding but not for diet (except for *mor103* and *mor124*), suggesting an essential role in the recognition of feeding cues in RT. Although previous studies have suggested that fish use olfaction for migration and homing [2], only a few putative MOR genes have been identified and their role remains purely speculative. Thus, we showed, for the first time, that two V1Rs (*ora1a* and *ora5b*), three V2Rs (*olfcg6*, *olfca1*, *olfch1*) and six MOR (*mor103*, *mor107*, *mor112*, *mor113 mor118* and *mor133*) genes should be considered for further examination of their potential role in the imprinting of nutrient detection olfactory cues. 

In parallel to the regulation of the olfactory receptor, our data demonstrated that dietary treatment could modify the olfactory epithelium (marker gene expression) from nasal to central system areas related to olfactory-driven behaviors, suggesting a modification in the transmission of olfactory cues into central nervous system circuitry. The difference of expression is limited (less than a two-fold change), suggesting little functional impact. Thus, olfactory signaling markers showed stability in the regulation of genes, suggesting strong tissue homeostasis in the three tissues studied.

Regarding neurotransmitters, 5-HT and DA are critical neuromodulators known to play a fundamental role in a multitude of cognitive processes. In fish, the telencephalon receives neuromodulatory inputs which could participate in the odor response [26]. In our experiment, higher L-Dopa levels were associated with the most palatable diet (C diet) before or after the feeding period. Therefore, higher levels of L-DOPA during the fasting period may increase the motivation to eat, but, in addition, during the feeding period when the L-DOPA level was higher than in the fasting period, once the smell had been associated with the reward Moreover, we found more 5-HT in the telencephalon of fish fed with the C diet. As for L-DOPA, higher levels of 5-HT during the fasting period may increase the motivation to eat, but not during the feeding period, when 5-HT level was lower than in the fasting period, suggesting that 5-HT level does not influence motivation to eat. Such findings suggest that neurotransmitters may be involved in olfactory cue responses and that their increase can promote appetite. 

Overall, these findings suggest that juvenile RT have a capacity for olfactory sensory perception that varies according to diet composition and nutritional status and may contribute to the perturbation in feeding behavior regulation and animal growth of RT fed with a vegetal diet at both peripheral and central levels. This knowledge could be important to promote the regulation of feed intake in farmed fish fed with modern sustainable aquafeed.

## 4. Materials and Methods

### 4.1. Animal Handling

Female RT fry used in this experiment originated from the same parental stock (INRAE Fish Farm of Lees-Athas, Permit number A64.104.1, vallée d’Aspe, France). The feeding experiment was conducted from first feeding to eight months in a recirculating rearing system at the INRAE facilities of Donzacq, France (authorisation number A40-228.1, Landes), and approved by the ethical committee (C2EA-73) of INRAE “Comité d’éthique Aquitain poissons oiseaux” (N°agreement INRAE 21699, 12 December 2019). All efforts were made to minimize fish numbers and suffering. All animals were used in the feeding trial. This experiment, which lasted a total of two years, made it possible to produce this article, but other analyses and samples were performed using other animals during this trial.

### 4.2. Experimental Diets

Diets were manufactured at the INRAE experimental facilities at Donzacq using a twinscrew extruder (Clextral). Pellet sizes were between 1 mm diameter and 1 mm length to 5 mm diameter and 5 mm length depending on RT size (1 mm for 0–2 months, 2/3 mm between 2–4 months, 4/5 mm between 4–8 months). The diets used throughout this trial were manufactured for two periods (0–2 g and 20 g—8 months) which contained the same raw materials between the same experimental diets. In order to adapt the formulation to different stages and fish size, the proportions of these ingredients were slightly different among diets used in the first and the second period of the trial. The experiment was conducted with one of two different experimental diets (Table S1): a C diet containing a mix of FM (27%), FO (12.1%) and plant ingredients, and a V diet, completely free from FM and FO, which were replaced by a blend of plant ingredients (10.8% sunflower oil, 5% palm oil and 2.7% linseed oil). The vegetal oil blend in the V diet was chosen in order to provide an overall amount of FA classes in similar proportions to those of the FA classes found in the C diet. For the V diet, DHA (present in FO for the C diet) was replaced with the equivalent amount of ALA by adding linseed oil (10.8%). 

Each of the two experimental diets contained 21.47% crude lipids (±0.27% of total diet) with the same amount of major *ω*-3 FA; 26.52% and 26.53% of DHA in C diet, 27.62% and 28.24% of ALA in V diet. This amount of *ω*-3 FA class was chosen in order to be close to the proportions of *ω*-3 FA classes found in the marine diet [9]. To obtain this amount of DHA in the C diet and to avoid contamination by EPA, a marine oil with concentrated DHA was used by adding (7.3%) Omegavie^®^ DHA oil (min 70%) with other FO (4.8%). The amount of lipids in others class of FA were closely similar between the two diets during all trials (Table S2). In order to avoid exceeding anti-nutrient threshold levels, we used a blend of wheat gluten, extruded peas and whole wheat, corn gluten meal, rapeseed meal and white lupin as protein sources (54.08% of total diet for the first period and 44.23% of the total diet for the second period). Synthetic L-lysine, L-methionine, dicalciumphosphate and soy-lecithin were added to all diets to correct deficiencies in essential amino acids, phosphorus and phospholipids. A mineral and vitamin premix was added to each diet. Diets were isoenergetic (*c.* 24.65 kJ g^−1^ of dry diet for all trials) and were formulated to cover the nutritional requirements of the RT [27]. 

Nutrient compositions of the diets were analyzed after drying the samples to constant weight at 105 °C for 24 h. Gross energy was determined in an adiabatic bomb calorimeter (IKA, Heitersheim Gribheimer, Germany). Starch content was evaluated by an enzymatic method (Megazyme). Ash content was determined by combustion in a muffle furnace (550 °C for 8 h). Crude protein was determined by the Kjeldahl method after acid digestion and the concentration was estimated by multiplying the nitrogen content using the 6.25 factor (see Table S3, for estimated amino acid composition). Crude lipids were quantified by the Soxhlet method using petroleum diethyl ether for the extraction as previously described [28]. Total lipids were extracted and measured gravimetrically according to the Folch method [29] using dichloromethane instead of chloroform. FA methyl esters (FAME) were prepared by acid-catalyzed transmethylation of total lipids using boron trifluoride (BF_3_) in methanol (14%) according to the Shantha and Ackman method [30] and analyzed in a Varian 3900 gas chromatograph (Varian, les Ulis, France) equipped with a fused silica DB Wax capillary column (30 m × 0.25 mm internal diameter, film thickness 0.25 μm; JW Alltech, Vire-Normandie, France). Injection volume was 1 μL, using helium as a carrier gas (1 mL/min). The temperatures of the injector and the flame ionization detector were 260 and 250 °C, respectively. The thermal gradient was as follows: 100–180 °C at 8 °C/min, 180–220 °C at 4 °C/min and a constant temperature of 220 °C for 20 min. FA were identified with reference to a known standard mixture (Sigma, St. Louis, MO, USA) and peaks were integrated using Varian Star Chromatography Software (Star Software, version 5, Walnut Creek, CA, USA). Individual FAs were expressed as a percentage of total FAME identified. 

### 4.3. Fish Rearing and Experimental Design

At the beginning of the experiment, RT fry with mean weight of 130 mg, were randomly distributed among 10 tanks (five tanks for each dietary treatment, 150 fish per tank). At the beginning of the trial, 50-L to 200-L tanks were used depending on the fish size (maximum stocking density: 22 kg/m^3^) to minimize tank size influence on feeding behavior [31]. Fish were exposed to natural photoperiod conditions (four seasons) and the water temperature was set at 15 ± 1 °C. During the trial, water dissolved oxygen was 9 mg L^−1^, ammonia < 0.01 mg L^−1^, nitrite < 0.04 mg L^−1^, and nitrate was about 17 ppm. The quantity of flow was 0.3 L/s by tank; all water of each tank was changed six times each hour. All fish were fed by hand twice a day with an interval of eight hours, until apparent satiety (feed intake parameters). Throughout the trial, dead fish (not differing between diet, less than 15%) were removed daily and weighed. At the end of eight months of feeding, either after five days fasting period or after a single meal, fish were first anaesthetized in a benzocaine bath at 30 mg L^−1^ and then killed in a benzocaine bath at 60 mg L^−1^. 

To determine the overall distribution, expression and regulation of the olfactory sensing pathways in RT induced by long-term diet exposure (eight months), RT were fasted for five days (first sampling). This allowed, firstly, evaluation of the long-term effect of the two diets while avoiding a meal effect (diet effect). Secondly, the fasted state enabled calculate of the basal level of receptor expression to study the postprandial effect (feed effect, 30 min after feeding, second sampling) of the two diets in RT. For this, ten olfactory rosette, olfactory bulb, and telencephalon brain tissue samples per condition (two fish per tank) were dissected and immediately frozen in liquid nitrogen and stored at −80 °C for qPCR analysis. A further ten telencephalon brain tissue samples were dissected for HPLC analysis. Two more fish per tank were randomly sampled, immediately frozen and kept at –20 °C for whole-body composition analysis. Prior to sampling, confirmation that the animals had consumed the feed was carried out. For zootechnical parameters, all animals were included (1500/1500 at the beginning of the trial). 

### 4.4. Variables and Analysis

Juvenile trout were counted and weighed every three weeks as a group from the beginning to the end of the trial (eight months). Variables related to growth were BWG per individual per day calculated during a period of three weeks and daily feed intake. Feed parameters were analyzed over the total feeding trial. Variables related to zootechnic parameters are presented in Figure 1.

### 4.5. Gene Expression Measurement by Real-Time Quantitative PCR

Total RNA was extracted from the olfactory rosette, olfactory bulb, telencephalon, (N = eight per fish) using the TRIzol^®^ reagent method (Invitrogen, Carlsbad, CA, USA) with Precellys^®^24 (Bertin technologies, Montigny le Bretonneux, France). Total RNA (2 μg) was used for cDNA synthesis. RNA concentration was tested by optical density (OD) using a NanoDrop 2000c (Thermo, Vantaa, Finland), and only samples with an OD 260 nm/280 nm ratio > 1.8 were used for analysis. The Super-Script III RNAse H-Reverse transcriptase kit (Invitrogen) was used with random primers (Promega, Chartonniéres-les-bains, France) to synthesize cDNA in a final volume reaction of 20 μL. Real-time [quantitative] PCR (RT-PCR) assays were performed with the Roche LightCycler480 system (Roche Diagnostics, Neuilly-sur-Seine, France). The reaction mix was 6 µL per sample, including 2 µL of diluted cDNA template (1:10), 0.12 µL of each primer (10 µmol L^−1^), 3 µL of Light Cycler 480 SYBR^®^ Green I Master mix and 0.76 µL of DNAse/RNAse-free water (5 Prime GmbH, Hamburg, Germany). The RT-PCR cycle was initiated at 95 °C for 10 min for the initial denaturation of the cDNA and hot-start Taq-polymerase activation, followed by 45 cycles of a two-step amplification program (15 s at 95 °C; 10 s at 60 °C). Melting curves were monitored systematically (temperature gradient 0.11 °C per second from 65 to 97 °C) at the end of the last amplification cycle to confirm the specificity of the amplification reaction. Duplicate wells were used for each sample and negative controls were included in all reactions consisting of wells containing RNA samples and water instead of cDNA. The efficiency of all qPCR reactions was 96–100% and R^2^ was 0.95–1. Data were extrapolated from standard curves and normalized to the housekeeping elongation factor 1α gene (*eef1α*) [32,33]. Relative expression of the target genes was determined by the ΔΔCT method [34]. Mean ± standard error of mean values for each group are expressed in fold changes relative to the C diet (olfactory rosette) for all genes in Figure 2, Figure 4 and Figure 6, *ora1a* gene C diet (olfactory rosette) for all genes in Figure 2, *olfcg6* gene C diet (olfactory rosette) for all genes in Figure 4, *mor103* gene C diet (olfactory rosette) for all genes in Figure 6 and C diet for all genes in Figure 8 and Figure 9. Accession numbers obtained and used in this study are available in NCBI and Ensembl genome browser. All gene sequences of RT used were identified by in silico analysis, as previously used in [32], from Genomicus software program, version 100.01 (www.genomicus.biologie.ens.fr) (Accessed on 21 February 2021) and Ensembl (http://www.ensembl.org, Ensembl Release 102; 1 November 2020, RT genome available) (Accessed on 21 February 2021) and queried against the human, mouse, salmon and zebrafish genome using the BLAST tool in Ensembl and in NCBI (https://blast.ncbi.nlm.nih.gov/Blast.cgi) (Accessed on 21 February 2021) to confirm gene identification. For paralog genes (such as *ora1* or *ora3*), the percentage of similar sequences was determined by alignment of the mRNA of RT genes using MUSCLE software (www.ebi.ac.uk/Tools/msa/muscle) (Accessed on 15 March 2021). In the case of genes which possessed numerous paralogs, when some of them presented highly similar sequences of over 95%, we built primer pairs to amplify paralogs together in order to not distinguish the expression of each of them (*mor103.2/103.3/103.4*, *mor129.1a/129.1b*, *olfcr1a/b* and *olfcv1/3*) (see Table S4).

### 4.6. Indolamine and Catecholamine Release Measurement by Ultra-High-Pressure Liquid Chromatography (UHPLC)

Catecholamines and indolamines in the telencephalon (*n* = 12) were separated and detected using a Waters^®^ Acquity H-Class Plus UHPLC System equipped with a thermostatted autosampler supported with a Waters^®^ Acquity Multi-λ Fluorescence Detector (Milford, MA, USA). Waters^®^ Empower™ Pro software was used for data acquisition and quantification. All reagents and standards were purchased from Sigma-Aldrich^®^ (Taufkirchen, Germany). All solvents used were molecular science grade. Each telencephalon was homogenized with Precellys^®^ tissue homogenizer in a 20 mM phosphate, 1 mM EDTA (pH = 6.5 ± 0.05) buffer. After centrifugation (14,000× *g*, 20 min, 4 °C), deproteinization of the supernatant was performed with a volume-to-volume 10% metaphosphoric acid solution. After centrifugation (14,000× *g*, 5 min, 4 °C), the supernatant was filtered with a 0.22 µm poly-vinylidene di-fluoride unit. Chromatographic separation was achieved on a Phenomenex^®^ PFP(2) column (4.6 mm × 150 mm i.d. 3 μm) at 30 °C. The injection volume was 10 μL and the flow rate was set at 0.4 mL/min. A quaternary solvent system was used, consisting of (A) pH 4.3 ± 0.05 10 mM phosphate buffer, (B) methanol, (C) ultrapure water and (D) acetonitrile. The mobile phase was filtered through in-line 0.2 μm membrane filters. The following gradient elution was employed: 0–11 min: 80% A, 20% B; 16 min: 50% C, 50% D; 16–26 min: 50% C, 50% D; 27 min: 80% A, 20% B and 27–35 min (column equilibration): 80% A, 20% B. The eluate was monitored using double excitation/emission fluorescence detection: 285 nm/355 nm for 5-HT and 5-HIAA; 228 nm/306 nm for L-DOPA and HVA. Metabolites were identified by comparing with standards. Quantification was based on integration of peak areas and compared to standard calibration curves (R^2^ correlation > 0.999) of each metabolite of interest. Calibration curves were linear from 0.01 to 3 pmol/injection for 5-HT, 5-HIAA, L-DOPA and HVA.

### 4.7. Statistical Analyses

All statistical analyses were performed using the R software (v3.6.1)/R Commander package (R Foundation for Statistical Computing, Vienna, Austria). Tanks were used as the experimental unit for data on growth parameters (Figure 1). Individual fish were the experimental unit for data on gene expression and indolamine/catecholamine release (Figure 2, Figure 3, Figure 4, Figure 5, Figure 6, Figure 7, Figure 8 and Figure 9), since no tank-related effect was observed during the experiment. Data are presented as mean ± standard error of the mean (SEM). Analyses were carried out on untransformed data as criteria for normality and homogeneity of variances were fulfilled (Shapiro–Wilk’s and Levene’s test, respectively). When both conditions were satisfied, a one-way ANOVA (*p*-value < 0.05) was performed to assess the effects of the diets on growth parameters, and on the distribution and expression of the olfactory receptor (Figure 2, Figure 4 and Figure 6), followed by a Tukey’s post hoc test (*p*-value < 0.05). Values for the mRNA level of transcripts related to olfactory and brain markers in the olfactory rosette, olfactory bulb and telencephalon were analyzed by *t*-tests (Figure 8). If the criteria (normality and homogeneity) were still not met, a non-parametric test was used for the analysis. Diet effect and feeding effect (Figure 3, Figure 5, Figure 7 and Figure 9) were analyzed using two-way ANOVA. If an interaction was detected (*p*-value < 0.05), data were finally analyzed using one-way ANOVA to test the diet effect and feed effect individually. A Tukey’s test was used as a post hoc test (*p*-value < 0.05).

## Figures and Tables

**Table 1:** In silico analysis of olfactory receptors found in the annotated RT genome assembly, Omyk_1.0.

	Human (*Homo sapiens*)	Mouse (*Mus musculus*)	Salmon (*Salmo Salar*)	Zebrafish (*Danio reno*)	Rainbow Trout (*Oncorhynchus mykiss*)
V1R-like (ORAS: olfactory receptors related to class A)	4	164	7 (2 with 2 paralogs)	6	3 (each 2 paralogs)
V2R-like (OFLCs: olfactory C family G protein-coupled receptors)	0	121	29	50	10 (2 with 2 paralogs)
MOR (Main olfactory receptor)	368	1037	11 (various paralogs, total gene 24)	37 (various paralogs, total gene 138)	14 (1 with 2 and 1 with 3 paralogs)

## Data Availability

The data are available on request from the corresponding author.

## Supplementary Materials

The following supporting information can be downloaded at: www.mdpi.com/article/10.3390/ijms23042123/s1.

## Abbreviations

5-HT	5-hydroxytryptamine, serotonin
5-HIAA	5-hydroxyindolacetic acid
ω-3 LC-PUFA	ω-3 long chain polyunsaturated fatty acid
ALA	alpha-linolenic acid; BWG, body weight gain
C	commercial-like diet
DA	dopamine
DHA	docosahexaenoic acid
EPA	eicosapentaenoic acid
FI	feed intake
FM	fish meal
FO	fish oil
GPCRs	G protein-coupled receptors
HVA	homovanillic acid
RT-PCR	real-time quantitative PCR
L-DOPA	L-3,4-dihydroxyphenylalanine
MOR	main olfactory receptor
OLFC	olfactory C family G-protein-coupled receptor, vomeronasal type 2 receptor-like
ORA	olfactory receptors related to class A, vomeronasal type 1 receptor-like
V	plant-based diet
RT	rainbow trout
UHPLC	ultra-high-pressure liquid chromatography
