# Supplementary material for: Detection and Modulation of Olfactory Sensing Receptors in Carnivorous Rainbow Trout (Oncorhynchus mykiss) Fed from First Feeding with Plant-Based Diet
Source: Int J Mol Sci. 2022 Feb 14;23(4):2123. doi: 10.3390/ijms23042123 (PMC8876700; doi:10.3390/ijms23042123)
Supplement: Supplementary file 1 [file ijms-23-02123-s001.zip › ijms-1535964-supplementary.pdf]

Table S1. Ingredients and composition of the experimental diets.

Diet	0 g–20 g		20 g–8 months	
**Ingredient (%)**	**Commercial-like (C)**	**Plant-Based Diet (V)**	**Commercial-like (C)**	**Plant-Based Diet (V)**
Fish meal	27.0	0.0	27.0	0.0
Extruded whole wheat	8.0	3.8	22.5	17.0
Corn gluten	13.0	27.2	8.7	17.0
Wheat gluten	17.4	29.0	7.0	17.0
Peas meal	3.0	2.46	3.0	6.0
Extruded Peas	7.0	6.23	7.0	9.5
White lupin seed meal	2.5	2.0	2.5	4.5
Rapeseed meal	0.6	3.5	0.8	3.0
Soy lecithin	0.0	2.0	0.0	2.0
L-Lysine	0.5	1.2	0.5	1.7
L-methionine	0.0	0.4	0.0	0.4
$CaHPO_4.2H_2O$	0.0	1.7	0.0	1.4
Mineral premix[a]	1.0	1.0	1.0	1.0
Vitamin premix[b]	1.0	1.0	1.0	1.0
Fish oil	4.8	0.0	4.8	0.0
Palm oil	2.0	5.0	2.0	5.0
Sunflower oil	4.9	2.7	4.9	2.7
Linseed oil	0.0	10.8	0.0	10.8
Omegavie® DHA marine oil (min 70%)[c]	7.3	0.0	7.3	0.0
Composition (% of dry matter)				
Dry matter (in % of diet)	96.15	94.25	96.75	96.79
Crude protein	53.76	54.4	44.38	44.07
Crude lipid	21.8	21.9	21.32	20.87
Starch	10.84	10.70	20.25	20.02
Ash	7.40	5.02	7.33	4.41
Energy (kJg-1 DM)	24.75	25.29	24.12	24.44

[a]Mineral premix: (g or mg kg^{-1} diet): calcium caRTonate (40% Ca), 2.15 g; magnesium oxide (60% Mg), 1.24 g; ferric citrate, 0.2 g; potassium iodide (75% I), 0.4 mg; zinc sulphate (36% Zn), 0.4 g; copper sulphate (25% Cu), 0.3 g; manganese sulphate (33% Mn), 0.3 g; dibasic calcium phosphate (20% Ca, 18% P), 5 g; cobalt sulphate, 2 mg; sodium selenite (30% Se), 3 mg; KCl, 0.9 g; NaCl, 0.4 g (UPAE, INRAE).
[b]Vitamin premix : (IU or mg kg^{-1} diet): DL-alpha tocopherol acetate, 60 IU; sodium menadione bisulphate, 5 mg; retinyl acetate, 15,000 IU; DL-cholecalciferol, 3000 IU; thiamin, 15 mg; riboflavin, 30 mg; pyridoxine, 15 mg; B12, 0.05 mg; nicotinic acid, 175 mg; folic acid, 500 mg; inositol, 1000 mg; biotin, 2.5 mg; calcium pantothenate, 50 mg; choline chloride, 2000 mg (UPAE, INRAE).
[c]Omegavie® DHA oil (min 70%) : concentrated marine oil produced mainly from anchovy and sardine oil. The crude oil is first refined, then purified and concentrated. This oil contains marine DHA Omega 3 fatty acids under Triglycerides form. From POLARIS, Quimper, France.

Table S2. Selected FA composition (% of total FA). Sum values are presented in bold.

	0 g–20 g		20 g–8 months	
**Fatty acids**	**Commercial-like (C)**	**Plant-Based Diet (V)**	**Commercial-like (C)**	**Plant-Based Diet (V)**
C12:0	0.10	0.18	0.13	0.22
C14:0	1.70	0.32	1.81	0.37
C15:0	0.03	0.04	0.23	0.03
C16:0	12.20	15.37	12.71	15.93
C17:0	0.22	0.07	0.23	0.07
C18:0	2.84	3.61	2.86	3.69
C20:0	0.31	0.23	0.30	0.24
C22:0	0.30	0.23	0.28	0.24
C24:0	0.10	0.14	0.04	0.12
Sum of saturated fatty acids	**18.07**	**20.20**	**18.60**	**20.88**
C16:1 ω-7	2.07	0.17	2.08	0.16

C18:1 ω-9	16.72	24.74	17.05	25.24
C20:1 ω-9	1.69	0.24	1.41	0.22
C22:1 ω-9	1.77	0.0	1.38	0.03
Sum of MUFAs	**22.30**	**25.15**	**21.92**	**25.65**
C18:2 ω-6 (LA)	20.30	26.34	20.45	24.84
C18:3 ω-6	0.13	0.0	0.08	0.0
C20:2 ω-6	0.17	0.0	0.17	0.0
C20:3 ω-6	0.11	0.0	0.12	0.0
C20:4 ω-6 (AA)	0.85	0.0	0.83	0.0
C22:2 ω-6	0.05	0.10	0.12	0.05
C22:4 ω-6	0.32	0.0	0.30	0.0
C22:5 ω-6	1.56	0.0	1.55	0.0
Sum of ω-6 LC-PUFAs	**24.09**	**26.61**	**23.62**	**24.90**
C18:3 ω-3 (ALA)	0.80	27.62	0.71	28.24
C18:4 ω-3	0.43	0.0	0.42	0.0
C20:3 ω-3	0.12	0.0	0.12	0.0
C20:4 ω-3	0.34	0.0	0.31	0.0
C20:5 ω-3 (EPA)	4.08	0.0	3.96	0.0
C21:5 ω-3	0.24	0.0	0.25	0.0
C22:4 ω-3	0.14	0.0	0.07	0.0
C22:5 ω-3	1.96	0.0	1.90	0.0
C22:6 ω-3 (DHA)	26.52	0.19	26.53	0.11
Sum of ω-3 LC-PUFAs	**34.67**	**27.86**	**34.25**	**28.36**
Sum of ω-3 (EPA + DHA)	**30.60**	**0.19**	**30.50**	**0.11**
ω-3 (DHA + EPA) / ω-6	**1.27**	**0.0**	**1.29**	**0.0**

Table S3. Selected amino acid in diet composition (estimated, % of dry matter).

	**0 g–20 g**		**20 g–8 months**	
**Amino Acids (% of Dry Matter)**	**Commercial-like (C)**	**Plant-Based Diet (V)**	**Commercial-like (C)**	**Plant-Based Diet (V)**
Alanine	2.93	2.92	2.37	2.22
Arginine	2.79	2.29	2.44	2.33
Aspartatic acid	4.09	3.41	3.47	3.11
Cysteine	0.67	0.84	0.5	0.64
Glutamatic acid	8.72	9.79	6.97	8.07
Glycine	2.35	1.95	1.95	1.62
Histidine	1.19	1.13	0.97	0.95
Isoleucine	2.3	2.2	1.88	1.93
Leucine	4.5	5.18	3.56	4.06
Lysine	2.92	2.35	2.75	2.91
Methionine	1.1	1.32	0.91	1.09
Phenylalanine	2.51	2.76	1.97	2.25
Proline	2.87	3.49	2.26	2.68
Serine	2.27	2.38	1.83	1.98
Threonine	1.7	1.36	1.5	1.25
Tryptophane	0.51	0.47	0.41	0.4
Tyrosine	1.67	1.76	1.37	1.44
Valine	2.5	2.36	2.05	1.99

Table S4. Nucleotide sequence of the PCR primers used to evaluate mRNA expression of transcripts by RT-qPCR.

Transcript	Forward Primer	Reverse Primer	Accession Number
**Reference**			
eef1a1	TCCTCTTGGTCGTTTCGCTG	ACCCGAGGGACATCCTGTG	AF498320
ORA Receptor			
ora1a	GTGGTCCAGTGGCTACATCC	GGGTGATCACTGTCTTGGCA	XM_021570507.1
ora1b	CGCTACCTGGCAGTGATCTT	TGCTGCATGTTGACCGAGTC	XM_021569534.1
ora3a	ACGGTGATTGGCGTGATCTT	CCTCAGAACAGACACCAGCA	XM_021611141.1
ora3b	GCTGGTGATGCTGTTTGTGG	ACAGAAGCAGAGAAGTGGGC	XM_021570503.1
ora5b	GCATAGTCAGAGCTCCACCC	AGGGCTGGTTGTTGTCTCTG	XM_021601370.1
MOR Receptor			
mor103 (103.2/103.3/103.4)	GGCC-TATCCTCTTCCTTACTGTG	AGCAGGAAAAC-TATAAGGAGCCC	FJ613854.1
mor107	CTGGCCTGCTCTGACATCTC	AACCACCACCACAGCATAGG	XM_021581807.1
mor109	CCTTCTGTGGCTCTCTGGTG	ACCAGGAAACAGACCCAAGC	XM_021619771.1
mor112	TAATATGTGGTTTGGCGGCG	TGAGTCAAACAGCTGGCCAA	XM_021581623.1
mor113	GGTTCTGAGGTTGGCATGGA	ACAGAGAGGGGCGAAGAGAA	XM_021581778.1
mor116	TCCCAAAGGTGCTGTCAGAC	ATAAAGAGAACCGCGCCTCC	XM_021581805.1
mor117	ACCCCAATGACCTACCCTGT	ACATGGGCTTGTGGAGAGTC	XM_021581744.1
mor118	GGGTGTATCGTTGATGGTGGT	TGTCTGAATGGCCTTGCTCC	XM_021587153.1
mor124	AGCCGTCAGACTTCCTTTGT	TCTGGTTGATGGTTATGGGTGA	XM_021586971.1
mor129 (129.1a/129.1b)	CAGCTCCTCTCCTCTGTCCT	TGGAAGGTGGAGGTGGAGAA	XM_021586987.1
mor133	ATCTCCCCTTTCAACCTGGC	CCACATGTGTCCTCCTGCAG	XM_021622508.1
mor134	TCGGCCGGTCTACTAGGAAA	ACACCAGCACCATGATCAGG	XM_021611114.1
mor135	TCAGCCAGAAAAGCCCAGAT	GCGGAAGTTGGGGAATAGGA	XM_021614394.1
OLFC Receptor			
olfcg6	CCTGGTCACCCCTACTAGCT	TGAAATGCCGTGTCCCAGAA	XM_021581770.1
olfch1	ATCCCAGGCCTCAGAGAGTT	GGAAAACCTGCAGCTGAACG	XM_021588500.1
olfcj1	GTCTGGTGGAGTTGTGGGAG	GTCTGTGTAGCGGGATCCAG	XM_021581601.1
olfcq9	GAGAGGACCTTTCAGTGGGC	AACGCGATCACCTAACCCTG	XM_021588490.1
olfcr1a/b	TACCGTGACCTAGGCCTCAA	AGGAGACAGAGAGAGGCCAG	XM_021581766.1
olfcu1	ACTCTCCTACCCCTCGTTCC	GGAGGTTCCCAAGATGCCAA	XM_021581767.1
olfcv1/3	GTCTGACCGTGTCCATCCTG	CAGGAAGCACAGAGTCAGGG	XM_021587119.1
olfcx3	CGAGGCCAAGTTCATCACCT	CGGCCACTGTGTACTTTCCT	XM_021588491.1
olfcc1	TGGGATGCGAGGGGTTTAAC	CATATCCCAGGTCCGTGTCG	XM_021581765.1
olfca1	CTTGTCTGTTTCGCCTGTGC	GGCACGAACATTACCCAGGA	XM_021592506.1
**Olfactory and Brain Markers**			
calb2a1	TCTGAAATGGCGAGGCTTCT	GCCGTTTCCATCCTTGTCGT	XM_021597940.1
calb2b1	ACTGGGCCTGTCTGAAATGG	AGGTCCTTCAGCAGTGCATC	XM_021607442
s100a	GAGTGCAATGGAGGGGCTAA	AAGTCACTCAGTTCGCCCTG	XM_021559274.1
s100b	CTTGATCAATGAGGAGCTGCC	CCAATTCCGAGTCTCCGTCC	XM_021608876.1
ompa	TTCTTGAACCCGTCGGAACA	ATTCGCTCCCCAAACTCCTG	XM_021587788.1
ompb	CCTCACCCACCTGATGAACC	TCTGCCTCATACTGGTGCAC	XM_021623885.1
pvalb5	GCAGAACTTCTCCTCAGGGG	AGCGCAGAACTCTTCCATCC	XM_021625755.1
pvalb8	CTGATGCCATAGACGCTGCT	AGACTTTCTTCACCTCCGCG	XM_021558676.1
rtor	AGCAATATGTTTCCCACTCAGA	ACATCAATGGGCAGGCTTTTG	NM_001124239.2
gna/olf1a	CGTTCAGCTCGCCAATCAAC	TCCTGCCAGAGAGTCTTTGC	NM_001129988.2
gna/olf1b	TTTCACTCTCTCGCGCTCTC	TGTGTGAGAGAGTGTGGTGC	XM_021564388.1
gna/olf2	GGAGGAGAAAGCACGCATCA	CACCAGCACCCAATAGCAGA	NM_001129989.1
**Catecholamine/Indolamine Metabolism**			
tph1a	ACACCAGAGCCAGACACATG	TCATCTGAAGCTCCGAGGGA	XM_021598622.1
tph1b	AGCGTCCGTTTACAGTGAGG	GCCCACGATGTCCAGTTCAT	XM_021598622.1
tph2	AGCACCTCAAAGACCACGTC	ACTGGTCGAGCTCTGCAATC	MG015698
5ht1aa	CCCAACACTCCACAGTCCTC	ACCGAGCGTCTTTACCGTTT	XM_021622104
5ht1ab	GAGGACCAACGGGGACCCGA	AATCGCCGTGCTTGACCGCA	CCAF0100015582
sert	CCTGCTGCCCTACATGTTGA	GGGGCAGATGTGTTTCCAGA	M_021582096.1
vmat2	TCGCCTTGCTGCTGGATAAT	GAGGCGGTGGTGTGATTCTT	XM_021609975.1

th	ACGCTCTCTCAAGGTGTTCG	AAAGTACTCCAGCCCCTCCA	XM_021564247.1
drd1	GGAGGAGCTGCAGAAGAAGG	TTTCCAGTGACACATCGGCA	XM_021617454.1
drd2	CCTCCAGTCCACCACCAATT	CCACTCTCCCACCACCTCTA	NM_001124372.2
dat	CTACCTCAGCGTCGACTTCC	TAGCACACCAAACCCGACTC	XM_021592557.1
